# New data on robustness of gene expression signatures in leukemia: comparison of three distinct total RNA preparation procedures

**DOI:** 10.1186/1471-2164-8-188

**Published:** 2007-06-22

**Authors:** Marta Campo Dell'Orto, Andrea Zangrando, Luca Trentin, Rui Li, Wei-min Liu, Geertruy te Kronnie, Giuseppe Basso, Alexander Kohlmann

**Affiliations:** 1 University of Padua, Laboratory of Molecular Diagnostic, Department of Pediatric Oncology, Via Giustiniani 3, 35128, Padua, Italy; 2 Roche Molecular Systems, Inc., Department of Genomics and Oncology, Pleasanton, CA, USA

## Abstract

**Background:**

Microarray gene expression (MAGE) signatures allow insights into the transcriptional processes of leukemias and may evolve as a molecular diagnostic test. Introduction of MAGE into clinical practice of leukemia diagnosis will require comprehensive assessment of variation due to the methodologies. Here we systematically assessed the impact of three different total RNA isolation procedures on variation in expression data: method A: lysis of mononuclear cells, followed by lysate homogenization and RNA extraction; method B: organic solvent based RNA isolation, and method C: organic solvent based RNA isolation followed by purification.

**Results:**

We analyzed 27 pediatric acute leukemias representing nine distinct subtypes and show that method A yields better RNA quality, was associated with more differentially expressed genes between leukemia subtypes, demonstrated the lowest degree of variation between experiments, was more reproducible, and was characterized with a higher precision in technical replicates. Unsupervised and supervised analyses grouped leukemias according to lineage and clinical features in all three methods, thus underlining the robustness of MAGE to identify leukemia specific signatures.

**Conclusion:**

The signatures in the different subtypes of leukemias, regardless of the different extraction methods used, account for the biggest source of variation in the data. Lysis of mononuclear cells, followed by lysate homogenization and RNA extraction represents the optimum method for robust gene expression data and is thus recommended for obtaining robust classification results in microarray studies in acute leukemias.

## Background

Microarrays have been demonstrated to be a powerful technology capable of successfully identifying novel taxonomies for various types of cancers [1-5] and gene expression signatures could also be associated with clinical outcome [2,4,6-9]. Those findings indicate that the data from different microarray assays are comparable enough to identify biological heterogeneity between distinct tumor types. Moreover, it has recently been demonstrated that, under properly controlled conditions, it is feasible to perform tumor microarray analysis, at multiple independent laboratories [10-15]. In addition, it has been shown that sample preparation by different operators did not impair the robustness of so-called diagnostic gene expression signatures [16]. To avoid possible sources of variation in the data, individual laboratories developed standardized protocols involving all the various steps of the sample preparation procedure, starting from tumor sample collection, through sample processing, total RNA isolation, cDNA synthesis, cRNA synthesis and labeling, target fragmentation, microarray hybridization, to washing and staining protocols. Users are recommended to use specific RNA isolation protocols, since one of the major concerns in microarray technology is the quality of starting material and various studies helped in a better understanding of the pre-analytical factors influencing gene expression signatures in peripheral blood and bone marrow [17,18]. However, until now, no fundamental information has been available about the degree of variation in the leukemia gene expression profiles resulting from different RNA extraction procedures although it is recognized that different RNA stabilization and isolation techniques will introduce varying amounts of analytical noise into the data [19-21].

**Figure 1:**
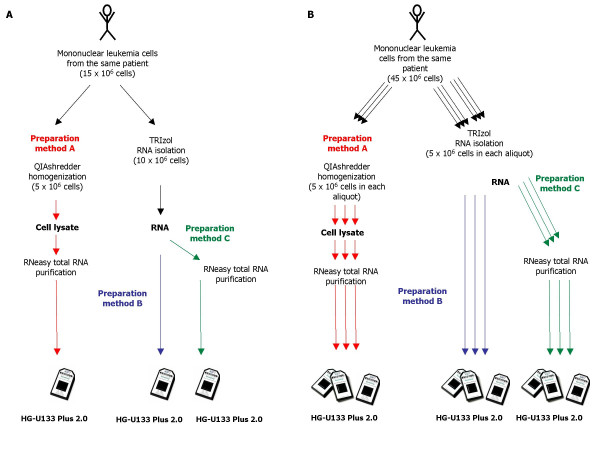
**Study concept**. (A) Total RNA of each of the first 24 samples had been extracted following three different total RNA purification methods A, B, and C. Method A: lysis of the mononuclear cells, followed by lysate homogenization (to reduce viscosity caused by high-molecular-weight cellular components and cell debris) using a biopolymer shredding system in a microcentrifuge spin-column format (QIAshredder, Qiagen) followed by total RNA purification (RNeasy Mini Kit, Qiagen). Method B: TRIzol RNA isolation (Invitrogen). Method C: TRIzol RNA isolation (Invitrogen) followed by an RNeasy purification step (RNeasy Mini Kit, Qiagen). The RNA purification step combines the selective binding properties of a silica-based membrane with the speed of microspin technology. It allows only RNA longer than 200 bases to bind to the silica membrane, providing an enriching for mRNA since nucleotides shorter than 200 nucleotides are selectively excluded. (B) For each of three additional samples, nine aliquots of mononuclear cells had been collected. Total RNA has been processed for each aliquot following one of the three methods and for each method three independent technical replicates were performed (A,A,A, B,B,B, C,C,C).

Here we present a comparative study of the microarray data using three different RNA isolation and purification techniques (HG-U133 Plus 2.0 microarrays, Affymetrix, Inc., Santa Clara, CA, USA). We have performed standardized experiments with total RNA extracted from pediatric acute leukemia patients to investigate whether different extraction protocols (see methods) result in comparable gene expression data from the same sample source (Figure 1A). Moreover, we assessed the variability between gene expression levels arising from multiple technical replicates of the same sample (Figure 1B). Leukemia gene expression signatures have been studied by numerous laboratories and have been proposed to have an application in a routine diagnosis workflow [22-25]. However, it is not clear, to what degree the various RNA isolation protocols impact the gene expression signatures due to method-related changes. We comprehensively addressed the question of RNA preparation for microarray analysis in leukemia and suggest a technique for introduction into routine laboratory diagnosis of pediatric acute leukemia by gene expression profiling.

## Results

### Assessment of data quality

In this study we first monitored data quality parameters. All gene expression profiles passed the quality filter and met our criteria for inclusion into further data analyses [see Additional File 2]. In detail, the cRNA yield was higher than 10.0 μg, the percentage of present called probe sets represented on the HG-U133 Plus 2.0 microarray is greater or equal to 20.0%, the scaling factor is below 10, the ratios of intensities of exogenous *Bacillus subtilis *control transcripts from the Poly-A control kit (*lys*, *phe*, *thr*, and *dap*) are greater or equal to 1, and the intensity ratio of the 3' probe set to the 5' probe set for the housekeeping gene *GAPD *is less than 3.0. Four samples showed a higher 3'/5' *GAPD *ratio (#25 method C, two preparations of #26 method B, #16 method B) but had otherwise acceptable quality parameters.

**Figure 2:**
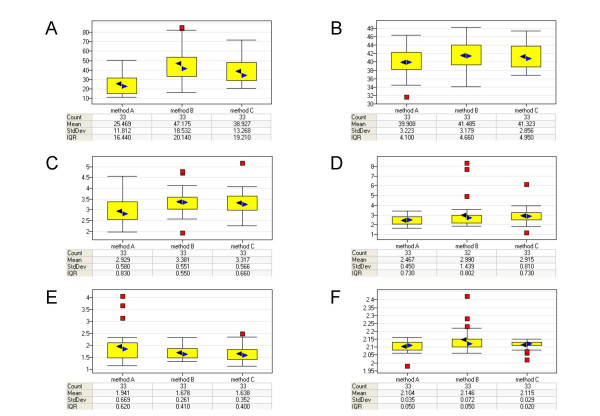
**Box plots of quality measurements**. The box plots show various quality metrics to judge overall performance of the microarray experiments. Each method represents 33 individual microarray experiments (Count). For each of the methods median values (blue arrow), mean values (black arrow), standard deviation (StdDev) and interquartile range (IQR) are given. The overall *p *value has been calculated for each of the parameters using one-way ANOVA. (A) total cRNA yield after in vitro transcription (A<C<B; *P *= 5,308e-^12^). (B) %P called transcripts (A<B, A<C, B~C; *P *= 0,020). (C) Scaling factor (A<B, A<C, B~C; *p *= 1,477e-^5^). (D) 3'/5' ratio of the housekeeping gene *GAPD*. Note: one sample was excluded in the *GAPD *box plot due to strong outlier behavior (PAD_00271, #16, TRIzol method). (E) Q value, defined as the average standard error of pixels in probe cells used for background computation (A>B, A>C, B~C; *P *= 0,0149). (F) the A260/A280 ratio of cRNA measured with a spectrophotometer (A<B, C<B, A~C; p = 0,00227).

As illustrated in Figure 2 the preparations of total RNA by QIAshredder homogenization followed by RNeasy purification (method A) resulted in acceptable cRNA yields and very reproducible low 3'/5' *GAPD *ratios. Preparations of total RNA by TRIzol (method B) yield slightly higher amount of cRNA, generate a lower image background as measured by Q value, but have a higher 3'/5' *GAPD *ratio. When the total RNA was prepared by TRIzol followed by RNeasy purification (method C) the cRNA yield was high, the background low, with the 3'/5' *GAPD *ratio being a little bit higher than for preparations of total RNA by QIAshredder homogenization followed by RNeasy purification. All three preparation methods generated an acceptable range of present calls on the whole genome microarray.

Total RNA quality can also be indirectly assessed by a so-called RNA degradation plot analysis as implemented in the "*Simpleaffy*" Bioconductor analysis package [26]. The sample degradation was consistently more severe in gene expression profiles when total RNA was processed for microarray analysis directly after isolation with TRIzol only (method B) [see Additional File 1, Supplementary Figure 1]. This might reflect that in method B more impurities such as phenol, salts, or residual ethanol are present in the starting total RNA as compared to method A or method C. These impurities influence the sample preparation reactions' efficiency, e.g. by inhibiting enzyme activities during cDNA synthesis or in vitro transcription reaction, and thus impair the microarray data generated with method B.

### Comparability of gene expression profiles

**Figure 3:**
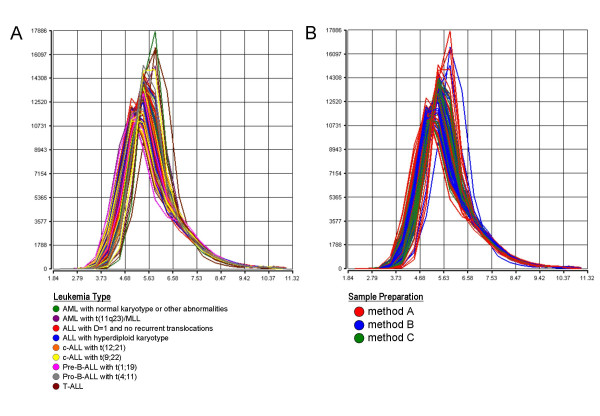
**Density curves of global signal intensities**. The plots show the overall signal density distribution of all probe sets represented on the HG-U133 Plus 2.0 microarray. The signal used is PS. Data from each microarray analysis is represented by a separate line. The plot is useful to visualize whether there are differences in the overall signal distributions of the experiments. (A) Density curves colored by nine distinct leukemia types. (B) Density curves colored by the three different sample preparation methods.

To assess the comparability of global gene expression data between samples isolated with different preparation methods it is useful to examine the overall signal distribution of all probe sets as density curve for each microarray experiment. Outlier experiments would be detected by their different behavior of the density curves. As shown in Figure 3A no substantial curve shifts in the microarray signal distribution are observed among samples representing different leukemia subtypes. The density curves are also overlapping when the signal distribution is plotted according to the total RNA preparation method (Figure 3B).

### Unsupervised data analysis

**Figure 4:**
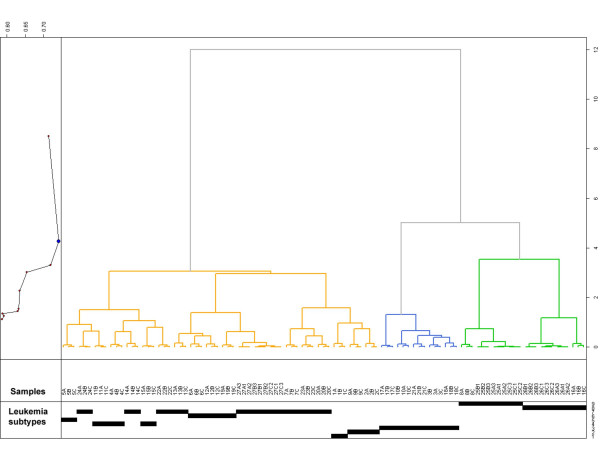
**Unsupervised hierarchical clustering analysis**. The unsupervised analysis is based on 2821 interquartile range (IQR) filtered probe sets of the HG-U133 Plus 2.0 microarray of the 99 experiments included in the study. The signal used is PQN. The three major clusters that were identified by the algorithm represent B lineage ALL (orange), T lineage ALL (blue). and AML (green) leukemia types. Then the dendrogram splits and samples are subdivided according to leukemia subtype characteristics: 1. Pro-B-ALL with t(4;11); 2. c-ALL with t(9;22); 3. T-ALL; 4. c-ALL with t(12;21); 5. Pre-B-ALL with t(1;19); 6. ALL with hyperdiploid karyotype; 7. c-ALL-Pre-B-ALL with DNA-Index DI = 1 and negative for recurrent translocations; 8. AML with t(11q23)/MLL; 9. AML with normal karyotype or other abnormalities. The graph on the left shows the correlation between distances for clustering validation (0–1-vector where 0 means same cluster, 1 means different clusters). Samples are labeled by patient numbers (#1 – #27) and total RNA extraction methods (method A, method B, or method C). For patient samples #25, #26, and #27, three individual technical replicates were performed.

We next investigated the consistency of gene expression measurements of leukemia samples when using different total RNA extraction methods by performing an unsupervised hierarchical clustering analysis. Expression data have been normalized using the PQN algorithm [27]. 2821 genes were selected using the interquartile range (IQR) as filtering criteria. The resulting dendrogram (Figure 4) clearly grouped the samples first by patient replicates using three different extraction methods and secondly separates the leukemias by lineage origin in B lineage ALL (orange), T lineage ALL (blue) and AML (green). In 22/27 of the patient replicates samples processed by QIAshredder homogenization followed by RNeasy purification (method A) cluster next to the two TRIzol-based purifications (method B, C). In 5/27 of triplets method A and C clustered next to method B (TRIzol without further purification). In no case did methods A and B together cluster next to method C. Within each lineage dendrogram the samples from the same leukemia subclasses are linked to each other. Within the B lineage cluster two patients with c-ALL with t(9;22) are linked together as well as two patients with hyperdiploid karyotype. Also, 3 patients with c-ALL with t(12;21) are linked in the same sub-branch. Patient samples with c-ALL-preB with DNA-index DI = 1 and negative for recurrent translocations are distributed over the three sub-branches of the B-ALL cluster. The latter may be interpreted as an illustration of the known heterogeneity within this subclass of acute leukemia. The group of T-ALL samples is not further subdivided. The cluster of myeloid leukemias is divided into two branches: AML with t(11q23)/MLL and AML with normal karyotype or other abnormalities. This clearly demonstrates that the underlying biology and not the RNA extraction protocol accounts for the biggest source of variation in the data. Also, in an unsupervised Principal Component Analysis (PCA) two distinct types of AML are clearly separated from T lineage ALL and from B lineage ALL and the three total RNA preparation methods for each patient sample can be found in close proximity next to each other [see Additional File 1, Supplementary Figure 2].

### Supervised data analysis

**Figure 5:**
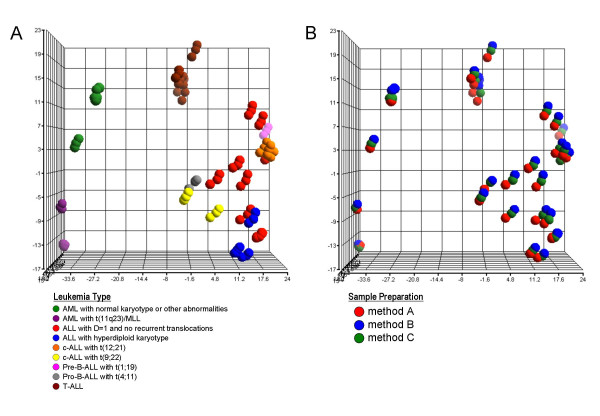
**Supervised analysis using differentially expressed genes**. In the three-dimensional principal component analysis (PCA) 99 samples are included. The signal used is PQN. The analysis is based on 1089 differentially expressed genes that were identified in a supervised way to distinguish between the 9 distinct leukemia subtypes. A sphere represents each sample's gene expression profile using the 1089-gene signature. The first three principal components (PC) account for 58.6% of variation of the data (PC1 = 40.3%, PC2 = 11.3%, PC3 = 7.01%). (A) Distinction by leukemia classification: spheres with the same colors represent the same leukemia subtype. (B) Distinction by sample preparation method: spheres with the same color represent samples processed with the same total RNA preparation method.

A supervised analysis was performed to assess the potential impact of the use of different total RNA extraction methods on a leukemia classification approach. An all-pairwise t-test analysis identified differentially expressed genes that would distinguish between the 9 classes of pediatric leukemias that are represented in our dataset. A gene set of 1089 differentially expressed probe sets was then examined by three-dimensional PCA. As shown in Figure 5A this gene set clearly separates the various leukemia lineages (B lineage ALL, T lineage ALL, AML) from each other. In the AML group t(11q23)/MLL positive samples are separated from AML with a normal karyotype or other abnormalities. In the B lineage ALL group subclusters can be identified for ALL with the recurrent translocations t(1;19), t(4;11), t(9;22), or t(12;21). Importantly, Figure 5B demonstrates that the three preparation methods for each patient sample can be found in close proximity next to each other. This again indicates that the data variability due to different preparation methods is less influential in the gene expression profiles than the leukemia subclass.

**Figure 6:**
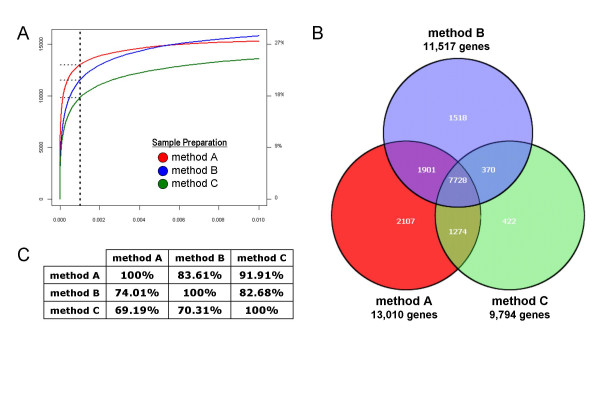
**One-way ANOVA of technical replicates**. Three patient samples (#25, #26, and #27) from distinct leukemia subtypes were analyzed in three independent technical replicates for each method A, B, and C leading to a dataset of 27 gene expression profiles. (A) The graph represents false discovery rate (FDR) values based on One-way Analysis of Variance (ANOVA) results. For each preparation method the absolute number (left *x*-axis) and percentage of differentially expressed genes (right *y*-axis) between the various leukemia subclasses is given. The *x*-axis is representing multiple percentages of false discovery rates (%FDR). Method A: red line, method B, blue line, method C, green line. The vertical line is drawn at a FDR of 0.001 (0.1%). (B) Venn diagram representing the absolute number of overlapping differentially expressed genes for the three methods used. The representation is based on a series of filters: present calls, fold-change, and FDR of 0.001 (0.1%). For example, n = 7,728 genes are found to be consistently differentially expressed between the various leukemia subclasses when comparing method A to B, method A to method C, and method B to method C. As a second example, n = 2,107 genes are exclusively found to be differentially expressed when using sample preparation method A. Alternatively, n = 1,274 genes are detected to be differentially expressed by both method A and method C, but not by method B. (C) Summary table representing the percentages of overlapping differentially expressed genes for the three methods used. The first line represents the comparisons of method A to method B or method C. The second line represents the comparisons of method B to method C or method A. The third line represents the comparisons of method C to method A or method C.

As shown in Figure 1B three patients had been analyzed with three technical replicates. To further assess the influence of total RNA preparation methods on a potential leukemia classification approach, an one-way analysis of variance (ANOVA) was performed separately for these technical replicates. For each method A, B, and C the absolute number of differentially expressed genes was identified using the following filtering strategy: (i) filtering by present calls, followed by (ii) filtering by fold-change, and (iii) filtering by false discovery rate (FDR). In detail, in the first filtering step for every probe set of 9 microarrays at each ANOVA at least 3 microarrays called the probe set as "present". In the second filtering step for every probe set at each comparison, i.e. #25 vs. #26, #25 vs. #27, and #26 vs. #27, the fold change is at least 1.5 fold. In the third filtering step the FDR cutoff was set as a threshold of 0.001. Then, the number of differentially expressed genes that are overlapping between the three methods was summarized. The analysis results are summarized in Figure 6. Figure 6A represents the FDR curves for the three different methods. At a FDR of 0.1% it can be observed that the absolute number of differentially expressed genes between the various leukemia subclasses is the highest when method A is performed (n = 13,010). The second highest number of differentially expressed genes is observed with method B (n = 11,517). The lowest number of differentially expressed genes is observed with method C (n = 9,794).

We next investigated the percentage of overlapping genes that are found to be differentially expressed between the three methods used when analyzing the various leukemia subclasses in a supervised way. The percentage of overlapping genes is another suitable parameter to address the impact of the use of different total RNA extraction methods on a leukemia classification approach. Figure 6B represents a Venn diagram visualization of the absolute number of differentially expressed genes that are overlapping between the three methods at a chosen false discovery rate (FDR) of 0.1%. In detail, n = 7,728 genes are found to be consistently differentially expressed between the various leukemia subclasses when comparing all three methods. Overall, comparisons of absolute numbers of differentially expressed genes of method A showed a greater overlap to the other methods than comparisons based on method B or method C, respectively. This can also be examined by percentages of overlapping differentially expressed genes between the three methods (Figure 6C). Again, at a chosen FDR of 0.1% the highest percentage of overlap is observed for method A. In detail, 83.61% of differentially expressed genes between the 9 leukemia subclasses are overlapping in the comparison of method A to method B. 91.91% of genes are commonly detected to be overlapping in the comparison of method A to method C. The second highest overlap is identified in the comparison of method B to method A (74.01%) and to method C (82.68%). Only 69.19% of differentially expressed genes are overlapping in the comparison of method C to method A, and 70.31% are overlapping in the comparison of method C to method B. Interestingly, n = 2,107 genes are exclusively found to be differentially expressed when using method A. An analysis where these 2,107 genes were annotated according to their biological function revealed that the top biological functions associated with these genes were cancer, cell cycle, cell signaling, DNA replication, recombination, and repair, gene expression, or RNA post-transcriptional modification [see Additional File 1, Supplementary Figure 3].

Additionally, to further illustrate the assay performance, a statistical power analysis for the RNA preparation methods A, B, and C is performed based on the Bioconductor package "ssize". The power analysis is used, for statistical comparison of identical leukemia samples, to assess the precision of technical replicates obtained from different RNA preparation methods. The data sets generated based on the preparations of total RNA following the methods A and B have greater average statistical power than the microarray data set based on method C [see Additional File 1, Supplementary Figure 4].

In summary, these analyses indicate that preparation of total RNA by QIAshredder homogenization followed by RNeasy purification is a robust sample preparation method for microarray experiments that outperforms other procedures for isolation of total RNA.

### Reproducibility and precision of different sample preparation methods

As three patients had been analyzed with three technical replicates (Figure 1B) we therefore were further able to assess the technical reproducibility and precision of gene expression data using the different total RNA extraction methods by examining squared correlation coefficients (R^2^), box plots, scatter plots, and coefficient of variation (CV) assessments. These analyses included all 54675 probe sets represented on the HG-U133 Plus 2.0 microarray.

**Figure 7:**
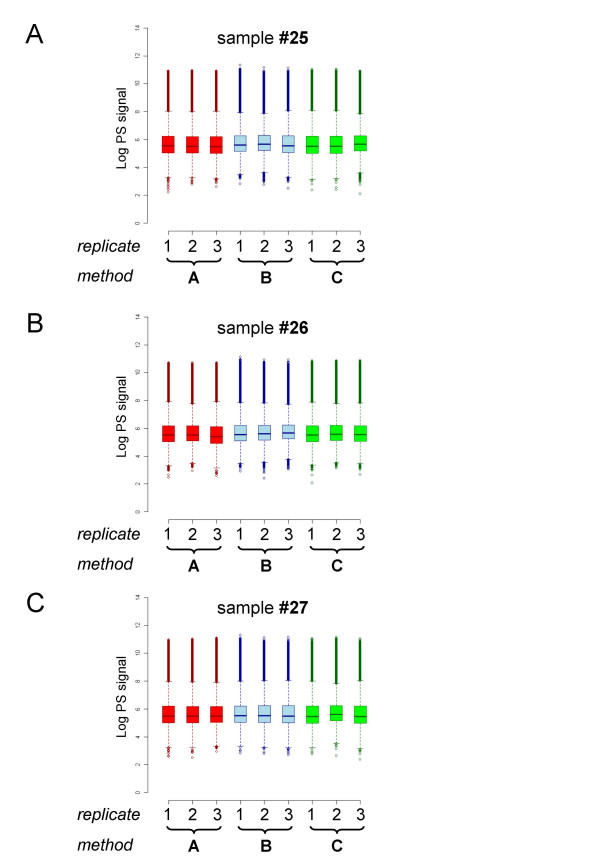
**Signal distributions for three technical replicates**. Individual signal intensity distribution on a probe set level (PS) are shown as box plots for the three technical replicates for each of the three methods used. Sample preparation types are pointed on the *x*-axes; the log value of PS signals are pointed on the *y*-axes. Box plots with the same color represent log value of PS signals from the same total RNA preparation procedure type method A (red), method B (blue), or method C (green), respectively. (A) Replicates of patient #25. (B) Replicates of patient #26. (C) Replicates of patient #27.

As shown in Figure 7, the mean values and interquartile ranges (IQR) of probe set level signals (PS) are highly comparable within the technical replicates as well as across three sample preparation methods. Furthermore, a pairwise scatter plot analysis demonstrates that gene expression data are well correlated within the three sample preparation methods [see Additional File 1, Supplementary Figures 5A,B,C]. The squared correlation coefficients R^2 ^range from 0.985 to 0.989 for preparations of total RNA by QIAshredder homogenization followed by RNeasy purification (method A), 0.976 to 0.987 for TRIzol isolation (method B), and 0.967 to 0.988 for TRIzol followed by RNeasy purification (method C). Between the three different sample preparation methods the mean value of R^2 ^is 0.952 and standard deviation is 0.005 for method A versus method B, 0.976 mean value and 0.005 standard deviation for method A versus method C, and 0.965 mean value and 0.011 standard deviation for method B versus method C, respectively.

Analysis of coefficient of variation is a useful way for assessment of reproducibility and precision of the gene expression profiles generated from three different total RNA sources. The box plots demonstrate the variability in gene expression measurements within the three technical replicates using different sample preparation methods [see Additional File 1, Supplementary Figure 6]. The data demonstrate that the sample replicates prepared with QIAshredder homogenization followed by RNeasy purification (method A) are tighter and more consistent across the three different subtypes of pediatric leukemia samples than those obtained with the other two RNA isolation methods. Also, it can be seen that microarray data generated with QIAshredder homogenization followed by RNeasy purification is least varied, most reproducible and precise. Supplementary Figure 7 [see Additional File 1, Supplementary Figure 7] represents the slopes in the scatter plots of the standard deviation versus the mean PS intensity signals calculated for each probe set on the HG-U133 Plus 2.0 microarray, referred to as robust CV (as described in the formula). Mean value and standard deviation of the slopes are 0.025 and 0.007 for method A, 0.052 and 0.017 for method B, 0.035 and 0.019 for method C.

## Discussion

Recent investigations successfully applied gene expression microarrays to classify known tumor types and also various hematological malignancies [5,25,28-34]. The increasing amount of data supports the concept that microarray analysis could be introduced soon into the routine classification of cancer [16,23,35]. However, several questions about the multitude of sources of variation in gene expression data have not been addressed and therefore continue to leave doubts about the performance of gene expression microarrays in clinical laboratory diagnosis. Here, for the first time, we present a study focused on analyzing the impact of different RNA preparation procedures on gene expression data for different subtypes of pediatric acute leukemias. The sample preparation and purification methods analyzed here are not only the three currently most used protocols for isolation of total RNA in laboratory diagnosis analyses but are also used by many laboratories working with different microarray platforms. The protocols examined are method A: lysis of the mononuclear cells, followed by lysate homogenization, which reduces viscosity caused by high-molecular-weight cellular components and cell debris, using a biopolymer shredding system in a microcentrifuge spin-column format, followed by total RNA purification; method B: TRIzol RNA isolation, and method C: TRIzol RNA isolation followed by a total RNA purification step using selective binding columns. The RNA purification step, based on selective silica-membrane, purifies all RNA molecules longer than 200 nucleotides consequently increasing the amount of mRNA. These three methods were analyzed in triplicates for each of 24 samples. Moreover, for an additional three samples triplicate technical replicates were performed for each protocol. The main purposes of this investigation were to address to what extent distinct total RNA template isolation techniques impair the precision and reproducibility of gene expression data from the same sample and secondly, whether the underlying characteristic leukemia-specific gene expression signatures are affected by the RNA preparation procedure. We finally aimed to identify the most robust sample preparation method for microarray experiments and, at the same time, a technique that could be introduced into daily routine laboratory practice.

After a first analysis of the quality of our microarray data, we could assert that since in all cases the quality parameters met our criteria, each of the three preparation methods is able to generate acceptable gene expression profiles of pediatric leukemias. We found that samples representing different leukemia subclasses and extracted using different RNA preparation methods are characterized by a high comparability of gene expression data thus demonstrating that sample preparation procedures do not impair the overall probe set signal intensity distribution. Importantly, even though yielding lower amounts of cRNA if compared to TRIzol (method B) and TRIzol followed by RNeasy (method C) protocols (A<C<B; *P *= 5,308e^-12^), the isolation of total RNA using QIAshredder homogenization followed by RNeasy purification (method A) resulted in a better quality of starting material as demonstrated by the A260/280 ratio of cRNA (A<B, C<B, A~C; p = 0,00227), by very reproducible low 3'/5' *GAPD *ratios, and by consistently lower scaling factors (A<B, A<C, B~C; *p *= 1,477e-^5^). This was then further examined by a so-called RNA degradation plot analysis as implemented in the *Simpleaffy *Bioconductor analysis package [26]. This analysis, although being an indirect approach for assessing the sample quality, demonstrated that the overall quality was consistently lower for microarray data when total RNA was processed for microarray analysis directly after isolation with TRIzol only (method B). While Agilent Bioanalyzer measurements showed acceptable total RNA quality profiles for all three methods the RNA degradation plot analysis might be a good way to indirectly identify poor quality samples via their global gene expression signatures on a probe level. The reason that total RNA samples prepared using method B demonstrate poor quality is probably due to the fact that impurities such as salts or residual amounts of phenol or ethanol are carried over in the sample preparation assay and subsequently impair enzymatic reactions.

Next, an unsupervised hierarchical clustering as well as unsupervised principal component analyses demonstrated that samples are grouped first by each patient's replicate method conditions, then by leukemia type, and finally by leukemia lineage. In fact, the B lineage ALL samples are all clustered together and separately grouped from T-ALL and AML. Moreover, inside each lineage-cluster leukemias with the same diagnostic features – e.g. recurrent translocations – are linked to each other. This finding is the demonstration that the variation in sample preparation method is a secondary effect, and that the major splits in the clusters reflect true underlying biological differences between leukemias.

These findings are then confirmed by a subsequent supervised analysis of gene expression data. Considering only the (n = 1,089) differentially expressed genes between the nine distinct leukemia categories that we studied here, all samples are clearly separated by leukemia lineages and without being influenced by the total RNA isolation method. Furthermore, AML with normal karyotype is separated from the two patient samples with AML with t(11q23)/MLL demonstrating an intra-lineage distinction within the AML group. The same separation can be observed in the B lineage ALL group where samples with the chromosomal aberrations t(1;19), t(4;11), t(9;22), or t(12;21) are split into distinct groups. As such, this is also an independent confirmation of the clustering organizations as presented in recent gene expression profiling studies of acute lymphoblastic leukemias [5,25,28,30-33,36].

## Conclusion

The first conclusion we draw from this study is that underlying biological characteristics of the pediatric acute leukemia classes are quite significant and largely exceed the variations between different total RNA sample preparation protocols. Having shown that at a chosen false discovery rate of 0.01% method A is producing a higher number of differentially expressed genes as compared to method B and method C, we would propose that lysis of the mononuclear cells, followed by lysate homogenization (QIAshredder) and total RNA purification (Qiagen) is the more robust total RNA isolation procedure for gene expression experiments using microarray technology. The importance of this new data is further strengthened by the analysis of the technical replicates. In fact, the gene expression data obtained with method A show the lowest degree of variation and are more reproducible, as compared to the alternative methods we tested for the isolation of total RNA. Finally, all these evidences, combined with the standardized microarray analysis protocol that we followed for this study let us conclude that the initial homogenization of the leukemia cell lysate followed by total RNA purification using spin columns is currently the optimal protocol available with respect to the robustness of gene expression data and that this method is practical for a routine laboratory use. Here we limited our microarray study to pediatric leukemia, but certainly these statements could also be applied to similar cohorts of adult leukemias.

## Methods

### Patient samples

Between December 2005 and March 2006 samples from twenty-seven acute pediatric leukemia patients were analyzed at the time of diagnosis. All patients received a laboratory diagnosis based on white blood cell count, cytomorphology, cytochemistry, multiparameter immunophenotyping, cytogenetics, fluorescence in situ hybridization (FISH), and molecular genetics (PCR). Chromosome aberrations t(1;19)(q23;p13)(*E2A-PBX1*), t(4;11)(q21;q23)(*MLL-AF4*), t(9;22)(q34;q11)(*BCR-ABL*) t(12;21)(p13;q22)(*TEL-AML1*), t(8;21)(q22;q22)(*AML1-ETO*), t(15;17)(q22;q21)(*PML-RARA*), inv(16)(p13;q22)(*CBFB-MYH11*), and t(8;14)(q24;q32) were screened following the BIOMED-1 concert action protocol [37]. Also, DNA index (DI) value analysis for all samples was performed to distinguish between patients with hyperdiploid karyotype and normal ploidy or hypodiploidy as reported by the Pediatric Oncology Group (POG) and Berlin-Frankfurt-Munster (BFM) group [38]. Patients with a DI value between 1.16 and 1.6 as detected by flow cytometry were considered hyperdiploid [38,39]. Based on the laboratory diagnosis, patients were subsequently risk stratified and enrolled in the AIEOP LAL-2002 or LAM-2002 protocols. This study was conducted after obtaining the informed consent from all patients following the tenets of the Declaration of Helsinki and was approved by the ethics committees of the participating institutions before the initiation of the study. All but one sample were drawn from bone marrow (BM). For one patient, an infant patient (age lower than one year; patient #26), a peripheral blood (PB) specimen was processed. Mononuclear cells from patients were isolated using Ficoll-Hypaque (Pharmacia-LKB, Uppsala, Sweden) density gradient centrifugation at our laboratory. For three myeloid cases (samples #8, #16, and #26) the specimens were processed by hemolysis. Both childhood acute myeloid leukemia (AML) (n = 4) and acute lymphoid leukemia (ALL) (n = 23) samples were collected (Table 1). The AML group included samples with t(11q23)/*MLL *rearrangement (n = 2; #16 is t(9;11) and #26 is t(1;11)) and AML patients with normal karyotype or other abnormalities (n = 2). The ALL group included Pro-B-ALL t(4;11) (n = 1), Pro-B-ALL/c-ALL with t(9;22) (n = 2), T-ALL (n = 5), c-ALL with t(12;21) (n = 3), Pre-B-ALL with t(1;19) (n = 1), B lineage ALL with hyperdiploid karyotype (n = 3), and B lineage ALL negative for the screened recurrent translocations and with a DNA index value equal to 1.0 (n = 8). The percentage of blast cells ranged between 70% and 98%.

**Table 1:** Patient characteristics, distribution, and total RNA preparation method

**Sample**	**Diagnosis**	**Blast cells (%)**	**Gold standard classification**	**Age (m-y)**	**RNA preparation method**	**Microarrays**
**#1**	Pre-pre-B-ALL	95	t(4;11)	6 m	A,B,C	3
**#2**	c-ALL	70	t(9;22)	14 y	A,B,C	3
**#3**	Early-T-ALL	83	negative	16 y	A,B,C	3
**#4**	c-ALL	90	t(12;21)	13 y	A,B,C	3
**#5**	Pre-B-ALL	98	t(1;19)	10 y	A,B,C	3
**#6**	c-ALL	87	hyperdiploid (DI = 1,233)	2 y	A,B,C	3
**#7**	c-ALL	93	negative	16 y	A,B,C	3
**#8**	AML	89	negative*	16 y	A,B,C	3
**#9**	c-ALL/Pro-B	85	t(9;22)	5 y	A,B,C	3
**#10**	Early-T-ALL	94	negative	4 y	A,B,C	3
**#11**	c-ALL	88	t(12;21)	2 y	A,B,C	3
**#12**	prepreB/c-ALL	96	Hyperdiploid (DI = 1,244)	11 y	A,B,C	3
**#13**	c-ALL	95	negative	3 y	A,B,C	3
**#14**	Pre-B-ALL	87	negative	12 y	A,B,C	3
**#15**	c-ALL	94	t(12;21)	8 y	A,B,C	3
**#16**	AML	93	t(9;11)	6 y	A,B,C	3
**#17**	Early-T-ALL	87	negative	9 y	A,B,C	3
**#18**	Early-T-ALL	93	negative	9 y	A,B,C	3
**#19**	c-ALL	93	hyperdiploid (DI = 1,160)	2 y	A,B,C	3
**#20**	prepreB/c-ALL	85	negative	3 y	A,B,C	3
**#21**	mature T-ALL	94	negative	4 y	A,B,C	3
**#22**	Pre-B-ALL	95	negative	7 y	A,B,C	3
**#23**	prepreB/c-ALL	94	negative	4 y	A,B,C	3
**#24**	c-ALL	86	negative	5 y	A,B,C	3

**TOTAL**						**72**

**#25**	AML	89	negative**	4 y	A,A,A,B,B,B,C,C,C	9
**#26**	AML	77	t(1;11)	6 m	A,A,A,B,B,B,C,C,C	9
**#27**	prepreB/CALL	92	negative	4 y	A,A,A,B,B,B,C,C,C	9

**TOTAL **(replicates)						**27**

**TOTAL in Study**						**99**

Negative = negative for all gold standard tests regarding recurrent translocations. Age at diagnosis is expressed as months (m) or years (y). Method A: QIAshredder homogenization using a biopolymer shredding system in a microcentrifuge spin-column format followed by RNeasy purification; Method B: TRIzol RNA isolation. Method C: TRIzol RNA isolation followed by RNeasy purification. Samples #1–24 have been extracted in triplicates; Samples #25, #26, and #27 have been extracted in multiple technical replicates (n = 3). (*) Karyotype information: 46, XY; (**) Karyotype information: 46, XY der(9p) [3]/46, XY [19].

### Study concept

As outlined in the study concept in Figures 1A and 1B 15 × 10^6 ^fresh mononuclear cells were collected for each of the first twenty-four leukemia samples (#1–24). Subsequently, total RNA was extracted from aliquots of 5 × 10^6 ^cells and 10 × 10^6 ^cells following two distinct total RNA purification method A and method B, respectively (see "RNA isolation for microarray analysis"). Total RNA obtained from method B was either used for the subsequent microarray analysis without further purification (method B), or was additionally purified following method C (see "RNA isolation for microarray analysis"). Microarray analysis was performed on each sample and each preparation method (Affymetrix HG-U133 Plus 2.0). Thus, for 24 patient samples a total of 72 microarrays were analyzed (Figure 1A). In three additional samples (#25–27) 45 × 10^6 ^fresh mononuclear cells each were collected and divided into nine aliquots of 5 × 10^6 ^cells. Again, total RNA was extracted from each aliquot following one of the three methods and for each method three technical replicates were performed (A,A,A, B,B,B, C,C,C), resulting in additional 27 gene expression profiles on Affymetrix HG-U133 Plus 2.0 microarrays (Figure 1B) [see Additional File 3].

### RNA isolation for microarray analysis

Mononuclear cells were processed immediately after or within 24 hours after the biopsy was obtained. Appearance and fluidity of the samples were monitored before starting with RNA isolation. Total RNA was isolated using three different methods. Method A: lysis of the mononuclear cells, followed by lysate homogenization using a biopolymer shredding system in a microcentrifuge spin-column format (QIAshredder, Qiagen, Hilden, Germany), followed by total RNA purification using selective binding columns (RNeasy Mini Kit, Qiagen). The cell lysate homogenization phase reduces viscosity caused by high-molecular-weight cellular components and cell debris. Method B: TRIzol RNA isolation (Invitrogen, Karlsruhe, Germany). Method C: TRIzol RNA isolation (Invitrogen) followed by a purification step (RNeasy Mini Kit, Qiagen). The RNA purification step previously mentioned combines the selective binding properties of a silica-based membrane with the speed of microspin technology. This system allows only RNA longer than 200 bases to bind to the silica membrane, providing an enriching for mRNA since nucleotides shorter than 200 nucleotides are selectively excluded. In all three methods we followed the protocols provided by the manufacturers. After extraction, total RNA was stored at -80°C until used for microarray analyses. RNA quality was assessed on the Agilent Bioanalyzer 2100 using the Agilent RNA 6000 Nano Assay kit (Agilent Technologies, Waldbronn, Germany). RNA concentration was determined using the NanoDrop ND-1000 spectrophotometer (NanoDrop Technologies, Inc., Wilmington, DE USA). The overall total RNA quality was assessed by A_260_/A_280 _ratio (NanoDrop) and electropherogram (Agilent Bioanalyzer).

### Microarray analysis

From each RNA preparation 2.0 μg of total RNA were converted into double-stranded cDNA by reverse transcription using a cDNA Synthesis System kit including an oligo(dT)_24 _– T7 primer (Roche Applied Science, Mannheim, Germany) and the Poly-A control transcripts (Affymetrix, Santa Clara, CA, USA). The generated cDNA was purified using the GeneChip Sample Cleanup Module (Affymetrix). Then, labeled cRNA was generated using the Microarray RNA target synthesis kit (Roche Applied Science) and an in vitro transcription labeling nucleotide mixture (Affymetrix). The generated cRNA was purified using the GeneChip Sample Cleanup Module (Affymetrix) and quantified using the NanoDrop ND-1000 spectrophotometer. In each preparation an amount of 11.0 μg cRNA were fragmented with 5× Fragmentation Buffer (Affymetrix) in a final reaction volume of 25 μl. The incubation steps during cDNA synthesis, in vitro transcription reaction, and target fragmentation were performed using the Hybex Microarray Incubation System (SciGene, Sunnyvale, CA, USA) and Eppendorf ThermoStat plus instruments (Eppendorf, Hamburg, Germany). Hybridization, washing, staining and scanning protocols, respectively, were performed on Affymetrix GeneChip instruments (Hybridization Oven 640, Fluidics Station 450Dx, Scanner GCS3000Dx) as recommended by the manufacturer.

### Image data analysis

Microarray image files (.cel data) were generated using default Affymetrix microarray analysis parameters (GCOS 1.2 software). Subsequently, intensity signals were calculated based on the non-central trimmed mean of Perfect Match intensities with Quantile Normalization [27]. For each gene expression profile a detailed data quality report has been generated to define the overall quality of each experiment [see Additional File 2]. The quality parameters that were monitored besides cRNA total yield and cRNA A_260_/A_280 _ratio included: (i) background noise (Q value), (ii) percentage of present called probe sets, (iii) scaling factor, (iv) information about exogenous *Bacillus subtilis *control transcripts from the Affymetrix Poly-A control kit (*lys*, *phe*, *thr*, and *dap*), and (v) the ratio of intensities of 3' probes to 5' probes for a housekeeping gene (*GAPD*).

### Statistical analysis

The data pre-processing included the summarization to generate probe set level signals for each microarray experiment and was performed using the PS or PQN algorithms as described elsewhere [27]. To analyze the quality and comparability of gene expression measurements we used a Quality Control (QC) matrix, density plots of scaled non-central trimmed mean of perfect match (PM) probe intensities (PS signal), and an unsupervised hierarchical clustering algorithm using Ward linkage of quantile normalized signals (PQN). To analyze the consistency of gene expression data we used a Principal Component Analysis (PCA) [40]. A subset of genes was selected using interquartile range (IQR) as filtering criteria and visualized by hierarchical clustering [41]. Data have further been analyzed using R software [42], Spotfire DecisionSite to generate the box plots [43], Ingenuity Pathways Analysis to annotate gene lists according to their biological function [44], and Partek Genomics Suite to generate signal density curves and PCA plots [45]. The power analysis was performed using the Bioconductor package "ssize" [46]. All microarray raw data are available through the Gene Expression Omnibus database, series accession number: GSE7757 [47].

## Competing interests

This study is part of the MILE Study (Microarray Innovations In LEukemia) program, an ongoing collaborative effort headed by the European Leukemia Network (ELN) and sponsored by Roche Molecular Systems, Inc., addressing gene expression signatures in acute and chronic leukemias. This study further supports the AmpliChip Leukemia Test program, a gene expression microarray test for the subclassification of leukemia. Roche Molecular Systems, Inc. has business relationships with Qiagen and is currently validating Qiagen products for the AmpliChip Leukemia Test.

## Authors' contributions

MCDO performed the microarray experiments and wrote the paper, LT contributed to perform the experiments, AZ, RL, and WML analyzed the microarray data, GB recorded clinical data, GK supervised the study and writing of the manuscript, and AK provided the original concept of the study, and contributed to writing the paper.

## Supplementary Material

Additional File 1 Supplementary Data. This file contains supplementary figures with additional comments explaining details of analysis, results, and interpretation. Click here for file

Additional File 2 This Excel file contains further details about each total RNA isolation method, including cRNA quality and quantity values as well as microarray quality and quantity values for each experiment. Click here for file

Additional File 3 This Excel file contains details about each total RNA isolation method and leukemia classification details for each CEL file. All microarray raw data (*.cel files) are available online through the Gene Expression Omnibus database with the series accession number GSE7757. Click here for file
