# Supplementary material for: New data on robustness of gene expression signatures in leukemia: comparison of three distinct total RNA preparation procedures
Source: BMC Genomics. 2007 Jun 22;8:188. doi: 10.1186/1471-2164-8-188 (PMC1925098; doi:10.1186/1471-2164-8-188)
Supplement: Additional File 1 — Supplementary Data. This file contains supplementary figures with additional comments explaining details of analysis, results, and interpretation. [file 1471-2164-8-188-S1.doc]

Supplementary Data

**Additional File 2. Data Quality Overview.** Parameters were monitored to assess the overall quality of each microarray experiment. Parameters related to sample preparation include cRNA yield and A260/A280 ratio after the in vitro transcription reaction. Parameters related to the microarray image after scanning and data processing include (i) Q value: defined as the average standard error of pixels in probe cells used for background computation, (ii) %P: percentage of present called probe sets, and (iii) SF: scaling factor. Parameters related to sample processing controls include the ratios of exogenous *Bacillus subtilis* control transcripts from the Affymetrix Poly-A control kit (*lys* (L), *phe* (P), *thr* (T), and *dap* (D)) and the ratio of intensities of 3’ probes to 5’ probes for the housekeeping gene *GAPD* [See *Additional File 2*].

**Supplementary Figure 1. RNA degradation analysis.** The box plot shows slopes from RNA degradation curves for each of the three methods as a function of 5’ to the 3’ position of the probes represented on the HG-U133 Plus 2.0 microarray. In general, high values for the slope are indicating a poor quality of starting total RNA as with RNA degradation or severe sample impurities the hybridized signals are systematically elevated at 3’ end compared to the 5’ end. The analysis is based on the function *AffyRNAdeg* in the *Simpleaffy* Bioconductor package [1]. Briefly, for each microarray experiment and within each probe set, individual perfect match (PM) probes for each probe set are arranged according to their proximity to the 5’ end location of the corresponding gene. The slope of averaged intensities can be computed as a linear function of probe positions and used as an overall data quality measure. A subsequent box plot analysis of the slopes helps to visualize the degree of sample quality. For each method a total of 33 preparations are given (Count), including the median values (blue arrow), mean values (black arrow), standard deviation (StdDev), and interquartile range (IQR), respectively. Red squares indicate outliers. More detailed information on the box-and-whisker plot analysis can be found online [2].

**Supplementary Figure 2. Unsupervised principal component analysis.** In the three-dimensional principal component analysis (PCA) 99 samples are included. The signal used is PQN. The analysis is based on the top 5000 genes that were identified in an unsupervised way according to the largest coefficient of variation. A sphere represents each sample’s gene expression profile using the 5000-genes signature. The first three principal components (PC) account for 43.7% of variation of the data (PC1=21.2%, PC2=13.1%, PC3=9.4%). (A) Distinction by leukemia types: spheres with the same colors represent the same leukemia subtype. Two distinct types of AML are clearly separated from T lineage ALL and from B lineage ALL. (B) Distinction by sample preparation method: spheres with the same color represent samples processed with the same total RNA preparation method. Also, in an unsupervised PCA the three total RNA preparation methods for each patient sample can be found in close proximity next to each other. This again indicates that the data variability is dominated by the leukemia subclass and less influenced due to different total RNA preparation methods.

**Supplementary Figure 3. Functional categories for exclusive method A genes.** The biological network analysis was performed as previously described with Ingenuity Pathways Analysis (Version 5.0), a web-based application that generates networks using differentially expressed genes from expression microarray data analyses [3]. Here, n=2,107 genes were further analyzed that were exclusively identified with method A. Briefly, a data set containing the n=2,107 gene identifiers in probe set format was uploaded as a tab-delimited text file into the Ingenuity Pathways Knowledge Base. Then each probe set was automatically mapped to its corresponding database gene object to designate so-called focus genes. Focus genes are genes from the analysis input data file that meet both of the following criteria: These genes have been designated as being of interest, i.e. they were identified to be differentially expressed in the method A one-way ANOVA after applying three different filtering criteria. Additionally, they directly interact with other genes (non-focus genes) in the Ingenuity global molecular network, which consists of direct physical, enzymatic, and transcriptional interactions between orthologous mammalian genes from the published, peer-reviewed content in Ingenuity’s Pathways Knowledge Base [4]. A total number of n=983 focus genes were used as the starting point for generating biological networks. To start building the networks, the application queries the Ingenuity Pathways Knowledge Base for interactions between focus genes and all other gene objects stored in the knowledge base, and generates a set of networks with a network size of 35 genes/gene products. The application then computes a score for each network according to the fit of the user’s set of significant genes. The score is derived from a p-value (Fischer’s exact test) and indicates the probability of the focus genes in a network being found together due to random chance. A score of 2 indicates that there is a 1 in 100 chance that the focus genes are together in a network due to random chance. Therefore, scores of 2 or higher have at least a 99% probability of not being generated by random chance alone. Biological functions are then calculated and assigned to each network. The top 10 networks are shown with 983 focus genes given in bold letters. Genes that are marked by asterisks were represented by multiple Affymetrix probe set identifiers in the input file.

**Supplementary Figure 4. Power curve analysis**. The power curves were generated based on the Bioconductor analysis package “ssize” [5]. Power is used to ensure a sufficient sample size is planned in order to achieve a high level of statistical power. The *x*-axis is representing the range of calculated power; the *y*-axis is representing the proportion of genes with a calculated power equal to or greater than a given power displayed on the *x*-axis. For each comparison (ANOVA), the power analysis for three total RNA preparation methods is performed to illustrate the assay performance. The results, based on gene-by-gene calculations, are shown in a cumulative plot of the fraction of genes detected as differentially expressed between the distinct leukemia subgroups, at a desired power for a given sample size (n=3). Method A and method B generate greater average powers as compared to method C.

**Supplementary Figure 5. Pairwise scatter plots for technical replicates.** Scatter plots of Log (PS) signal intensities from all genes represented on the HG-U133 Plus 2.0 microarray are given for each of the three technical replicates and three sample preparation methods, respectively [6]. Each diagonal panel represents the label for method A, B, or C as well as the number of the technical replicate 1, 2, or 3. Each lower panel represents squared Pearson correlation coefficient values of R^2^. Each composite panel is referring to a single technical replicate: (A) Patient #25. (B) Patient #26. (C) Patient #27.

**Supplementary Figure 6.** **Coefficient of variation (CV) values for technical replicates.** Representation of %CV values using PS signals within technical replicates using different sample preparation methods. (A) Sample preparation types are pointed on the *x*-axis, signal intensity values are given on the y-axis. Each plot represents the global gene expression data from three technically replicated experiments. Box plots with the same color represent PS signals from the same total RNA preparation procedure method. (B) Table of box plot statistics, including mean values, interquartile ranges (IQR), as well as values for quartiles one (Q1) and three (Q3).

**Supplementary Figure 7. Trellis scatter plots of signals within technical replicates.** The Trellis scatter plots are showing the slopes of the standard deviation (StdDev) values (*y*-axis) versus the mean values of PS signals among the three technical replicates (*x*-axis). Data points are given for each probe set on the HG-U133 Plus 2.0 microarray according to the different sample preparation methods A, B, and C and patients: Patient #25 (upper panel), patient #26 (middle panel), and patient #27 (lower panel). The analysis, referred to as robust CV, has been performed as reported by Chudin and colleagues (described in the formula). Mean value and standard deviation of the slopes are 0.025 and 0.007 for method A, 0.052 and 0.017 for method B, and 0.035 and 0.019 for method C.

**Supplementary Figure 1. RNA degradation analysis.**

**
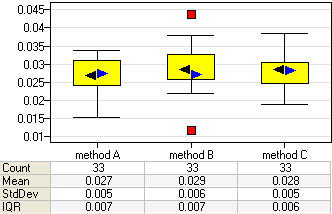
**

**Supplementary Figure 2. Unsupervised principal component analysis.**

**Supplementary Figure 3. Functional categories for exclusive method A genes.**

**
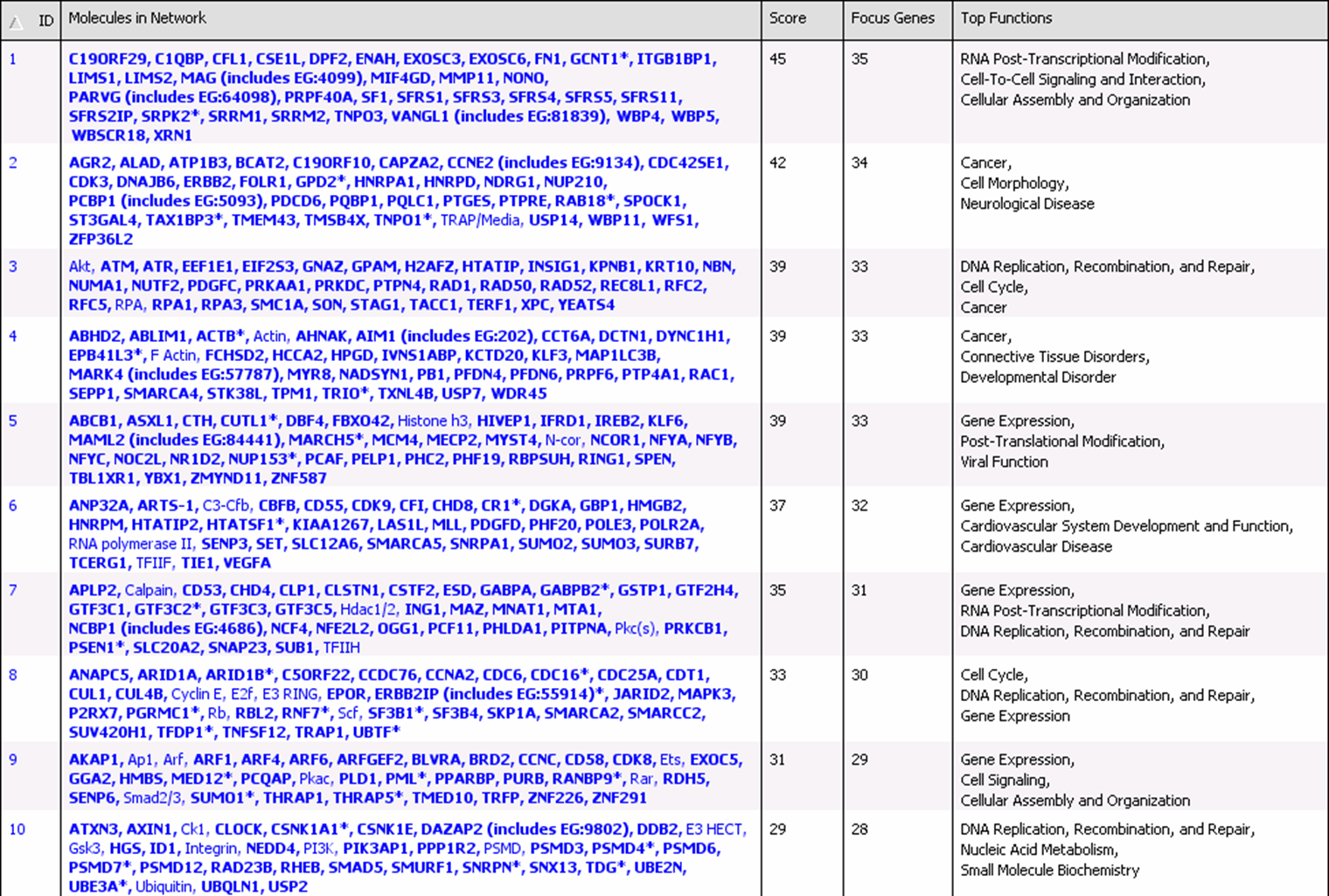
**

**Supplementary Figure 4. Power curve analysis**.

**Supplementary Figure 5A. Pairwise scatter plots for technical replicates.**

**Scatter Plots for Patient ID: #25**

**Supplementary Figure 5B. Pairwise scatter plots for technical replicates.**

**Scatter Plots for Patient ID: #26**

**Supplementary Figure 5C. Pairwise scatter plots for technical replicates.**

**Scatter Plots for Patient ID: #27**

**Supplementary Figure 6.** **Coefficient of variation (CV) values for technical replicates.**

**A Box plot graphs**

**B Table of box plot summary statistics**

| ***patient*** | ***#25*** | | | ***#26*** | | | ***#27*** | | |
| --- | --- | --- | --- | --- | --- | --- | --- | --- | --- |
| ***method*** | ***A*** | ***B*** | ***C*** | ***A*** | ***B*** | ***C*** | ***A*** | ***B*** | ***C*** |
| **Mean** | 1.311 | 1.688 | 2.229 | 1.718 | 1.841 | 1.363 | 1.423 | 1.421 | 2.008 |
| **IQR** (Q3-Q1) | 1.154 | 1.434 | 1.894 | 1.552 | 1.433 | 1.231 | 1.248 | 1.208 | 1.784 |
| **Q1** | 0.568 | 0.810 | 1.144 | 0.765 | 0.976 | 0.572 | 0.627 | 0.643 | 0.945 |
| **Q3** | 1.722 | 2.244 | 3.037 | 2.318 | 2.409 | 1.803 | 1.875 | 1.852 | 2.729 |

**Supplementary Figure 7. Trellis scatter plots of signals within technical replicates.**
